# Therapeutic Application of *Dendrobium fimbriatum* Hook for Retinopathy Caused by Ultraviolet Radiation and Chemotherapy Using ARPE-19 Cells and Mouse Retina

**DOI:** 10.3390/plants13050617

**Published:** 2024-02-23

**Authors:** Chi-Feng Cheng, Sheue-Er Wang, Chen-Wen Lu, Thi Kim Ngan Nguyen, Szu-Chuan Shen, Chia-Ying Lien, Wu-Chang Chuang, Ming-Chung Lee, Chung-Hsin Wu

**Affiliations:** 1 School of Life Science, National Taiwan Normal University, Taipei 116, Taiwan; draculachi@yahoo.com.tw (C.-F.C.); kumo.lu@gmail.com (C.-W.L.); marynguyen@ntnu.edu.tw (T.K.N.N.); scs@ntnu.edu.tw (S.-C.S.); 2 Department of Oncology, Taipei City United Hospital, Renai Branch, Taipei 106, Taiwan; 3 Department of Pathological Inspection, Saint Paul’s Hospital, Taoyuan 330, Taiwan; se.wang@msa.hinet.net; 4 Master Program of Sport Facility Management and Health Promotion, National Taiwan University, Taipei 116, Taiwan; chiayinglien@ntu.edu.tw; 5 Sun Ten Pharmaceutical Co., Ltd., New Taipei City 231, Taiwan; cwctd331@sunten.com.tw; 6 Brion Research Institute of Taiwan, New Taipei City 231, Taiwan; mileslee@sunten.com.tw

**Keywords:** *Dendrobium fimbriatum*, retinopathy, chemotherapy, UV, oxidative stress, inflammation, apoptosis, ARPE-19 cells, mice

## Abstract

Retinopathy caused by ultraviolet radiation and cancer chemotherapy has increased dramatically in humans due to rapid environmental and social changes. Therefore, it is very important to develop therapeutic strategies to effectively alleviate retinopathy. In China, people often choose dendrobium to improve their eyesight. In this study, we explored how *Dendrobium fimbriatum* extract (DFE) protects ARPE-19 cells and mouse retinal tissue from damage of ultraviolet (UV) radiation and chemotherapy. We evaluated the antioxidant capacity of DFE using the 1,1-diphenyl-2-trinitophenylhydrazine (DPPH) assay. The protective effects of DEF from UV- and oxaliplatin (OXA)-induced damage were examined in ARPE-19 cells using 3-(4,5-Dimethylthiazol-2-yl)-2,5-diphenyltetrazolium bromide (MTT) assay and immunofluorescence (IF) stains, and in mouse retinal tissue using immunohistochemistry (IHC) stains. Our results show that DFE has excellent antioxidant capacity. The ARPE-19 cell viability was decreased and the F-actin cytoskeleton structure was damaged by UV radiation and OXA chemotherapy, but both were alleviated after the DFE treatment. Furthermore, DFE treatment can alleviate OXA chemotherapy-induced reduced expressions of rhodopsin and SOD2 and increased expressions of TNF-α and caspase 3 in mouse retinal tissue. Thus, we suggest that DFE can act as suitable treatment for retinopathy through reducing oxidative stress, inflammation, and apoptosis.

## 1. Introduction

Retinopathy is an increasingly important public health problem due to an aging population and increased longevity of life [1]. Retinal pigment epithelium (RPE) cells are primarily responsible for cell structure maintenance and nutrient supply to maintain the health and function of photoreceptors. Oxidative stress can trigger RPE dysfunction, and atrophic changes in the RPE are a key feature of the progression of age-related macular degeneration [2]. Recently, ultraviolet (UV) radiation has increased significantly because of a gradual disappearance of the ozone layer in the atmosphere, exacerbating the damage of UV radiation to the human retina. As a result, the occurrence of retinopathy-related diseases has increased significantly [3]. With the rapid development of society and economy, eating habits have gradually changed. Unhealthy eating habits and excessive sugar intake have led to the occurrence of diabetes and cancer. Patients with long-term diabetes are prone to have inflammation and chronic vascular damage in the retina, which can even lead to blindness in severe cases [4,5].

According to data in 2020, there were 19.3 million new cases of cancer and nearly 10 million deaths worldwide [6]. That is to say, 1 in 5 people will develop cancer in their lifetime; 1 in 8 men and 1 in 11 women will die from cancer. Although chemotherapy could improve the survival rates of cancer patients, the adverse effects of the treatments may seriously impair long-term functional status, work productivity, and quality of life. For example, platinum chemotherapy drugs such as cisplatin, carboplatin, and oxaliplatin are widely used in the clinical treatment of various cancers. However, these platinum chemotherapy drugs may cause severe hearing loss [7]. Cisplatin-induced hearing loss is particularly harmful in children. Even mild hearing impairment can adversely affect academic and social development [8]. Approximately 500,000 patients diagnosed with cancer each year in the United States may be candidates for cisplatin treatment [9]. Cisplatin increases the risk of hearing damage or ototoxicity by 5-fold. More than half of adult and pediatric cancer patients treated with cisplatin develop hearing impairment, which has a significant impact on patients’ health-related quality of life [10]. Furthermore, it has been reported that platinum chemotherapy drugs such as oxaliplatin (OXA) not only cause peripheral neuropathy but also induce retinopathy in some patients [11,12]. OXA is a third-generation platinum-based chemotherapy drug used in the first-line treatment of advanced colorectal cancer [13]. The side effects of OXA treatment are ocular toxicity and neurotoxicity. Ocular toxicity is transient and reversible but neurotoxicity is a prominent side effect [14]. Clinical evidence has confirmed that patients undergoing OXA chemotherapy may develop retinopathy, visual loss, and other symptoms [15]. 

Whether retinopathy results from UV radiation, diabetes, or cancer chemotherapy, it is always caused by oxidative stress and inflammation in retinal tissue. Actively searching for natural medicines that can relieve oxidative stress and inflammation is very important for preventing and treating retinopathy. In China, traditional Chinese medicine (TCM) has been used for the alleviation of retinopathy for thousands of years [16]. Among these TMCs, dendrobium is often chosen to enhance the production of body fluid and improve eyesight. The main biological components of dendrobium are polysaccharides, alkaloids, bibenzyl, phenanthrenequinone, etc. These components have been reported to have neuroprotective, anti-cancer, immunomodulatory, antioxidant, platelet aggregation inhibition, and anti-aging activities [17]. Polysaccharides isolated from dendrobium were found to have antioxidant and anti-hyperglycemic activities [18]. Many studies have found that dendrobium can reduce diabetic retinopathy by preventing oxidative stress and inflammation in the retina [19,20]. *Dendrobium fimbriatum* Hook, one of dendrobium species, is a famous and valuable TCM. *D. fimbriatum* polysaccharides can exhibit a variety of biological activities, such as immune regulatory, hypoglycemic, and anti-oxidative effects [21,22]. Therefore, it is possible that *D. fimbriatum* can act as suitable treatment for retinopathy.

In this study, we selected *Dendrobium fimbriatum* extract (DFE) as an experimental material to explore the therapeutic effect of DFE on retinopathy caused by UV radiation and OXA chemotherapy. We used ARPE-19 cells and mouse retinas as experimental models. Our experimental results show that DFE has good antioxidant and anti-inflammatory effects and can be suitable for the treatment of retinopathy.

## 2. Materials and Methods

### 2.1. Preparation of the Dendrobium Fimbriatum Extract (DFE)

In this study, the DFE was selected as a TCM for alleviating chemotherapy-induced retinopathy. The *Dendrobium fimbriatum* was propagated and cultivated into plants using tissue culture technology by Sun-Ten Pharmaceutical Company (New Taipei City, Taiwan). The DFE was extracted and prepared from the stems of *Dendrobium fimbriatum*. The DFE preparation method was as follows: We pre-extracted the dried stem powder with 95% ethanol at 25 °C for 24 h, stirred it, and then reacted it with distilled water at 80 °C for 2 h. The extracts were precipitated with ethanol at a final concentration of 80% (*v*/*v*) for 24 h, and the proteins were removed using the Sevag method. The alcohol-insoluble material was then freeze-dried for 48 h to obtain DFE.

### 2.2. Determination of Chromatographic Fingerprinting of the DFE

The determination of chromatographic fingerprinting of the main active compounds of DFE was entrusted by HERBIOTEK Company (New Taipei City, Taiwan). Briefly, HERBIOTEK Company used high-performance liquid chromatography (HPLC, SHIMADZU LC 20-A) to perform chromatographic fingerprint analysis of all compounds of the DFE that were dissolved in distilled water/methanol. The active compound of DFE was inferred by comparing the retention time of each peak with that of an actual control. HERBIOTEK Company used denchrysan A, erianthridin, dendroflorin, moscatilin, confusarin, gigantol, dengibsin, chrysotoxin, moscatin, erianin, and chrysotobibenzyl as analysis index components and the purity of all the reference standards was above 98%. Conditions of HPLC were summarized as follows: (a) column: waters/sunfire RP18, 5 µm, 150 mm × 4.6 mm ID; (b) mobile phase: (A) 0.05% TFA aqueous solution, (B) acetonitrile; (c) detection: PDA (λ = 220 nm); (d) injection volume: 10 µL; (e) analytic concentration: 0.4 mg/mL; (f) oven temperature: 30 °C, (g) run time: 70 min.

### 2.3. DPPH Assay of the DFE

We used α,α-diphenyl-β-trihydrazide (DPPH) to detect the anti-free radical and antioxidant capabilities of DFE. First, we placed DFE at different concentrations (0.1, 0.5, 1, 5, 10 mg/mL) in a 96-well plate and then evenly mixed with 100 μL of 1.5 mM/mL DPPH (D9132, Sigma-Aldrich Co., St. Louis, MO, USA). After incubation at room temperature for 30 min, we used a microplate spectrophotometer (μQuant, Biotek Intruments, Inc., Winooski, VT, USA) to record the absorbance value at a wavelength of 517 nm. In addition, we also recorded appropriate blank treatments and standards (L-ascorbic acid; A5960, Sigma-Aldrich) as controls. We used the following formula to calculate the DPPH scavenging activity (%) of DFE: 100 × [(DFE + DPPH absorbance) − (DFE blank absorbance)]/[(DPPH absorbance) − (methanol absorbance)].

### 2.4. Experimental Design

**Figure 1:**
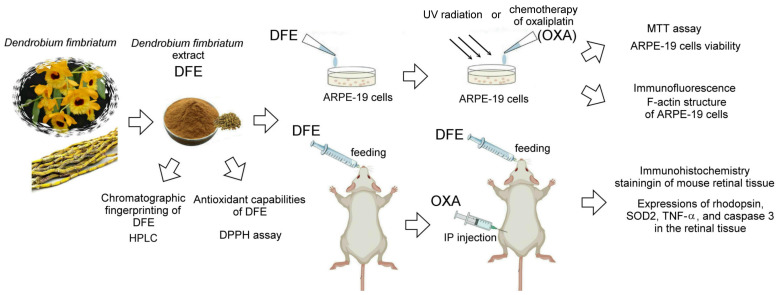
Schematic experimental protocol using ARPE-19 cells and BALB/c mice as cell and animal models, respectively, to study the therapeutic application of DFE in retinal damage caused by ultraviolet radiation and chemotherapy. DFE: *Dendrobium fimbriatum* extract; OXA: oxaliplatin.

The schematic experimental protocol for studying the therapeutic application of DFE in retinal damage caused by ultraviolet radiation and chemotherapy using ARPE-19 cells and BALB/c mice as cell and animal models, respectively, is shown in Figure 1. In this experiment, 3-(4,5-Dimethylthiazol-2-yl)-2,5-diphenyltetrazolium bromide (MTT, M5655, Sigma–Aldrich) was used to examine the protective effect of the DFE on the injury of ARPE-19 cells caused by UV radiation and chemotherapy of oxaliplatin (OXA, TTY Biopharm Co., Ltd., Taipei, Taiwan). Immunofluorescence was used to examine the damage of F-actin structure in ARPE-19 cells caused by chemotherapy of oxaliplatin. In the animal experiment, 9 BALB/c mice (aged 4 months) were divided into sham group (*n* = 3), oral DFE treatment group (DFE, *n* = 3), and oral DFE and OXA chemotherapy treatment group (DFE + OXA, *n* = 3). The DFE and DFE + OXA groups were treated with DFE (150 mg/kg) twice a day for two weeks. The dose of DFE was mainly selected for cell and mouse experiments based on the optimal dose that shows the best antioxidant capacity using the DPPH assay. The sham group were fed ad libitum. During the third week, the DFE + OXA groups were administered intraperitoneal injections of OXA (2.4 mg/kg) every other day for two weeks, and the sham and DFE groups were administered intraperitoneal injections of saline solution at the same time. After the fourth week, all mice were sacrificed after anesthesia, and then the eye tissues were collected for further immunohistochemical (IHC) staining.

### 2.5. ARPE-19 Cells Preparation

In this experiment, we used ARPE-19 cells to study the therapeutic application of DFE in retinal damage caused by ultraviolet radiation and chemotherapy. ARPE-19 cells possess the structural and functional properties of human RPE cells, providing important evidence for in vitro research on retinal damage. ARPE-19 cells were purchased from the Hsinchu Food Industry Development Research Institute Cell Bank (FIRDI, Cat. No.: BCRC-60383). We prepared the culture medium for ARPE-19 cells that contains high-glucose DMEM (Gibco BRL, Grand Island, NY, USA), 10% FBS, 1.5 g/L sodium bicarbonate, 0.11 g/L sodium pyruvate, 4 mM L-gluten, amino acid, 100 U/mL penicillin, and 100 μg/mL streptomycin. We maintained ARPE-19 cells in a humidified incubator at 37 °C and 5% CO_2_, and then we used a scraper to collect the ARPE-19 cells and moved them into a 24-well culture plate. We adjusted the density of ARPE-19 cells to 1 × 10^5^ cells per well for subsequent experiments. In this study, a total of 5 × 10^5^ cells/mL ARPE-19 cells were added to a 24-well plate, and 0, 0.1, 0.5, 1, 5, and 10 mg/mL of DFE were added to ARPE-19 cells. Then, ARPE-19 cells were divided into sham, UV, and OXA groups. Those ARPE-19 cells of the sham group were cultured in a humidified incubator at 37 °C and 5% CO_2_. Those ARPE-19 cells of the UV group were exposed under a custom-designed UV irradiation unit at 37 °C and 5% CO_2_. UV exposure of the ARPE-19 cells was conducted 10 cm from the source at 365 nm with the intensity of ~0.06 J/cm^2^/s for 30 min. For minimizing absorption of radiation by the medium, a thin layer of medium was retained above the cells throughout the UV exposure. Those ARPE-19 cells of OXA group were added to 10 μg/mL OXA (TTY Biopharm) followed by culturing within 24 h in a humidified incubator at 37 °C and 5% CO_2_.

### 2.6. MTT Assays of ARPE-19 Retinal Cells

We added 0.5 mg/mL of 3-(4,5-Dimethylthiazol-2-yl)-2,5-diphenyltetrazolium bromide (MTT) solution (M5655, Sigma-Aldrich Co., St. Louis, MO, USA) into ARPE-19 cells of sham, UV, and OXA groups, and then we cultured the ARPE-19 cells for 2 h and removed the supernatant. After adding 100 μL/well of DMSO organic solvent followed by 5 min shaking, absorbance measurements were taken at 570 nm.

### 2.7. Immunofluorescence of Cytoskeleton Using ARPE-19 Retinal Cells

In this study, we used rhodamine phalloidin dye R-415 (Thermo Fisher Scientific, Waltham, MA, USA) for cytoskeleton labeling, and then used 4′,6-diamidino- 2-phenylindole, dihydrochloride (DAPI) dye (Thermo Fisher Scientific) for nuclei labeling in ARPE-19 retinal cells. We viewed and analyzed fluorescent images of cytoskeleton and nuclei in ARPE-19 retinal cells using Leica DM IRB inverted fluorescence microscope and Leica Application Suite software (LAS) V4.12 (Wetzlar, Germany) individually. We visualized the rhodamine-labelled cytoskeleton of ARPE-19 retinal cells by red fluorescence that was excited at 540 nm and detected the dye emission at 565 nm. We visualized DAPI-labelled nuclei of ARPE-19 retinal cells labeled by blue fluorescence that were excited at 358 nm and detected the dye emission at 461 nm. 

### 2.8. Animal Preparation

As for animal experiments, BALB/c mice were purchased from the National Laboratory Animal Center (NLAC; Taipei, Taiwan). The animal experiment protocol was approved by the Institutional Animal Care and Use Committee of National Taiwan Normal University (Project No. 110026). Animal experiments were conducted in accordance with the International Guide for the Care and Use of Laboratory Animals, and animal experiments were considered to comply with the 3R principles (replacement, reduction, and improvement) to optimize experimental design. All BALB/c mice used in this experiment were housed in the animal facility of National Taiwan Normal University at 22 °C ± 2 °C with a 12 h light/dark cycle, and the BALB/c mice had ad libitum access to water and food.

### 2.9. IHC Staining Analysis Using Mouse Retinal Tissue

BALB/c mice were anesthetized and perfused with PBS containing 4% formaldehyde (EM grade glutaraldehyde solution, Sigma-Aldrich Corporation). Eye tissue samples from BALB/c mice were fixed with 4% formaldehyde (Sigma-Aldrich) and embedded in paraffin. Eye tissue specimens were cut into 5 μm thick sections by using a tissue microtome, and then sections were mounted on glass slides. Some eye tissue sections were stained with hematoxylin and eosin (H&E) (Sigma-Aldrich Corporation) to assess tissue integrity. Other eye tissue sections were subjected to IHC staining with antibodies of rhodopsin (Cat. numbers ab221664; Abcam, Waltham, Boston, USA), SOD2 (Cat. numbers ab110300; Abcam), purified rat antimouse tumor necrosis factor (TNF)-α (Cat. numbers #3707; Cell Signaling Technology, Danvers, MA, USA), and caspase 3 (Cat. numbers #3623; Cell Signaling Technology) for 1 h at room temperature, respectively. Sections were incubated with biotinylated secondary antibody (NovolinkTM Polymer Detection System l, Leica Biosystems Newcastle Ltd., Newcastle, UK) for 30 min and avidin–biotin–horseradish peroxidase (HRP) complex (Novolink™ Polymer Detection System l, Leica Biosystems Newcastle Ltd.) for additional 30 min. Immunostaining was visualized by using DAB Chromogen (NovolinkTM Polymer Detection System 1, Leica Biosystems Newcastle Ltd.) and slides were counterstained with hematoxylin (NovolinkTM Polymer Detection System 1, Leica Biosystems Newcastle Ltd., Benton Ln, Newcastle, UK). By means of Image J software 1.x (NIH, Bethesda, MD, USA), the relative number of cells in the outer nuclear layer (ONL) and inner nuclear layer (INL) within the retinal tissue of BALB/c mice were counted by adjusting the threshold when the image converted to 8 bit and then were measured to obtain the overall area. The protein expressions of rhodopsin, SOD2, TNF-α, and caspase 3 within the retinal tissue of BALB/c mice were obtained from the image of IHC staining by adjusting parameters such as color, saturation, and brightness, and then we calculated an area with uniform colors. 

### 2.10. Statistical Analysis

In this study, SigmaPlot 12.5 (Systat Software Inc., Chicago, IL, USA) was used for data analysis and chart production. Each group of experiments was repeated at least three times, and all experimental values were expressed as mean values ± standard error of the mean (SEM). Differences between the groups was tested using one-way or two-way ANOVA, followed by the Student–Newman–Keuls multiple comparison post hoc tests. A *p*-value less than 0.05 was considered statistically significant.

## 3. Results

### 3.1. HPLC Fingerprint of the Dendrobium Fimbriatum Extract (DFE)

**Figure 2:**
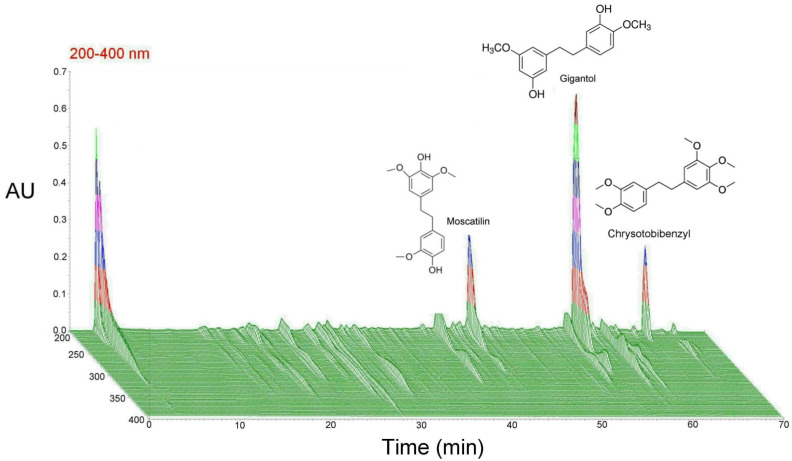
The HPLC fingerprint of the *Dendrobium fimbriatum* extract (DFE). The main active compounds of the DFE were moscatilin, gigantol, and chrysotobibenzyl. The chemical structures of the active compounds are shown in the upper blank space. Color of peaks represents differences in data scale.

In Figure 2, index compounds of DFE samples were identified by comparing the HPLC retention times of standard components. Under the HPLC chromatographic conditions, we established that the retention times of possible index components in the DFE HPLC chromatogram are very similar to those of the standard. Using Denchrysan A, erianthridin, dendroflorin, moscatilin, confusarin, gigantol, dengibsin, chrysotoxin, moscatin, erianin, and chrysotobibenzyl as standard components, we found that the main active compounds of DFE were moscatilin, gigantol, and chrysotobibenzyl using the HPLC fingerprint in Figure 2. 

### 3.2. Antioxidant Capacity of the DFE

**Figure 3:**
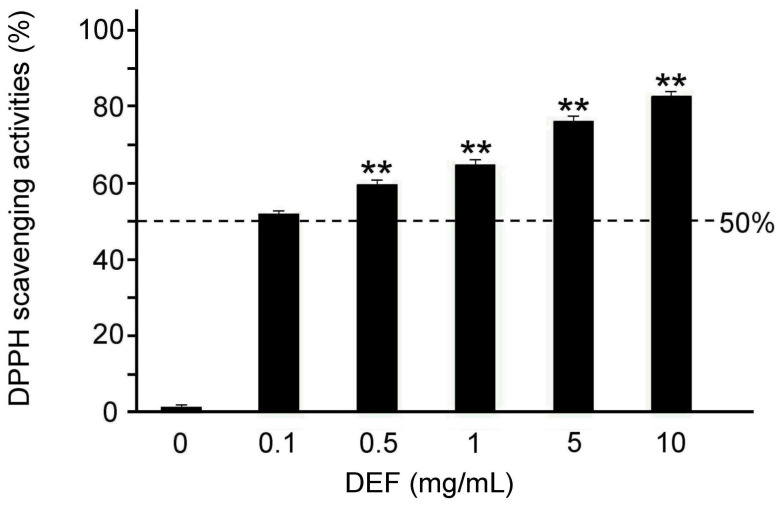
Antioxidant capacity of DFE treatments. Quantified DPPH free radical scavenging activities are significantly increased with concentrations of DFE treatments. (*n* = 3 for each group, ** *p* < 0.01, one-way ANOVA followed by Student–Newman–Keuls multiple comparison post test).

With the DPPH assay, we found that the free radical scavenging activity was gradually increased with the dose of DFE treatment (Figure 3). The result showed that the free radical scavenging activities were greater than 50% when concentrations of DFE treatments were 0.5, 1, 5, and 10 mg/mL and significantly better than the concentrations of DFE treatments at 0.1 mg/mL (Figure 3, *p* < 0.01). In other words, DFE treatments have a better antioxidant capacity which is able to possibly mitigate or prevent oxidative stress damage.

### 3.3. DFE Treatment Alleviates UV Radiation Damage in ARPE-19 Cells

**Figure 4:**
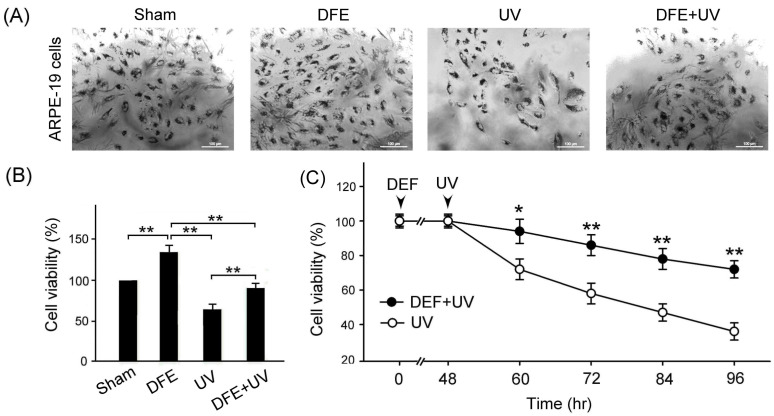
DFE treatment alleviates UV radiation-induced damage in ARPE-19 cells. (**A**) ARPE-19 cell staining with treatments of sham, DFE, UV radiation, or DEF + UV radiation. Scale bar = 100 μm. (**B**) Quantified ARPE-19 cell viability with DFE treatment significantly greater than those cells with sham and UV radiation damage (*p* < 0.01, DEF vs. sham, UV, and DEF + UV). Under UA radiation damage, DFE treatment significantly alleviated the reduction in ARPE-19 cell viability (*p* < 0.01, DEF + UV vs. UV). (**C**) Quantified ARPE-19 cell viability with UA radiation was gradually decreased over time, but DFE treatment significantly alleviated the reduction in ARPE-19 cell viability caused by UV radiation damage (*p* < 0.01–0.05, DEF + UV vs. UV). Data are shown as mean ± SEM (*n* = 3 for each group, ** *p* < 0.01, * *p* < 0.05, one-way ANOVA followed by Student–Newman–Keuls multiple comparison post tests).

With the MTT assay, we examined the effects of DFE treatment on ARPE-19 cell viability under sham and UA radiation damage. Our results showed that UA radiation damage obviously decreased ARPE-19 cell viability (Figure 4A, Sham vs. UV), and DFE treatment obviously alleviated the reduction in ARPE-19 cell viability under UA radiation damage (Figure 4A, DEF + UV vs. UV). We quantified ARPE-19 cell viability with DFE treatment that was significantly greater than that of ARPE-19 cells with sham and UV radiation damage (Figure 4B, DEF vs. Sham, UV, and DEF + UV, *p* < 0.01). Under UA radiation damage, DFE treatment can significantly alleviate the reduction in ARPE-19 cell viability (Figure 4B, DEF + UV vs. UV, *p* < 0.01). 

We further examined and quantified ARPE-19 cell viability after UA radiation damage over time. Our results showed that the ARPE-19 cell viability with UA radiation was gradually decreased over time after UA radiation damage (Figure 4C, UV), but DFE treatment can significantly alleviate the reduction in ARPE-19 cell viability caused by UV radiation damage (Figure 4C, DEF + UV vs. UV, *p* < 0.01–0.05). Our results revealed that DFE treatments may potentially provide therapeutic protection in ARPE-19 cells under UV radiation damage.

**Figure 5:**
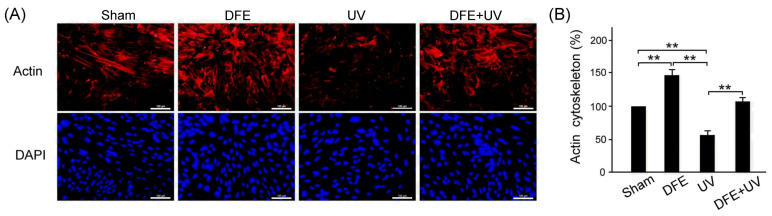
DFE treatment protects the morphology of F-actin structures in ARPE-19 cells under UV radiation damage. (**A**) Rhodamin-phalloidine (red) and DAPI (blue) staining of ARPE-19 cells with treatments of sham, DFE, UV radiation, or DEF + UV radiation. Scale bar = 100 μm (**B**) Quantified IF expression of F-actin among ARPE-19 cells with treatments of sham, DFE, UV radiation, or DEF + UV radiation. Data are shown as mean ± SEM (*n* = 3 for each group, ** *p* < 0.01, one-way ANOVA followed by Student–Newman–Keuls multiple comparison post tests).

By immunofluorescence (IF) staining assay, we examined the effects of DFE treatment on the morphology of F-actin structures in ARPE-19 cells under sham treatment and UV radiation damage. Our results showed that DFE treatment obviously enhanced IF expression of F-actin in ARPE-19 cells (Figure 5A, DFE), UV radiation obviously decreased IF expression of F-actin in ARPE-19 cells (Figure 5A, UV), and DFE treatment obviously alleviated the reduction in IF expression of F-actin in ARPE-19 cells under UV radiation (Figure 5A, DFE + UV). We quantified IF expressions of F-actin in ARPE-19 cells with DFE treatment that were significantly greater than those ARPE-19 cells with sham treatment and UV radiation damage (Figure 5B, DEF vs. Sham, UV, and DEF + UV, *p* < 0.01). Under UA radiation damage, DFE treatment significantly alleviated the reduction in IF expressions of F-actin in ARPE-19 cells (Figure 5B, DEF + UV vs. UV, *p* < 0.01). The results revealed that a DFE treatment can potentially protect the morphology of F-actin structures in ARPE-19 cells under UV radiation damage.

### 3.4. DFE Treatment Alleviates OXA Chemotherapy Damage in ARPE-19 Cells

**Figure 6:**
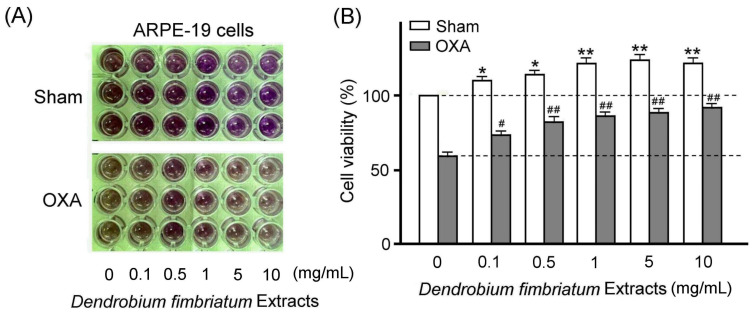
DFE treatment alleviates OXA chemotherapy-induced damage in ARPE-19 cells. (**A**) Effects of the DFE on ARPE-19 cell viability under sham and OXA chemotherapy damage by MTT assay. (**B**) Quantified ARPE-19 cell viability was significantly increased with concentrations of DFE treatments (*p* < 0.01–0.05) but was significantly reduced after OXA chemotherapy damage (*p* < 0.01). Furthermore, ARPE-19 cells with OXA chemotherapy damage can significantly increase with concentrations of DFE treatments (*p* < 0.01–0.05). Data are shown as mean ± SEM (*n* = 3 for each group, ** *p* < 0.01, * *p* < 0.05, ^##^ *p* < 0.01, ^#^ *p* < 0.05, two-way ANOVA followed by Student–Newman–Keuls multiple comparison post tests).

With the MTT assay, we examined the effects of DFE treatment on ARPE-19 cell viability under the sham treatment and OXA chemotherapy damage. Our results showed that 0.1–10 mg/mL DFE treatment obviously increased ARPE-19 cell viability (Figure 6A, Sham), but OXA chemotherapy damage obviously decreased ARPE-19 cell viability (Figure 6A, OXA). We further observed that DFE treatment can obviously alleviate the reduction in ARPE-19 cell viability under OXA chemotherapy damage (Figure 6A, OXA). We quantified ARPE-19 cell viability that was significantly increased with concentrations of DFE (Figure 6B, Sham, *p* < 0.01–0.05) but was significantly reduced after OXA chemotherapy damage (Figure 6B, OXA, *p* < 0.01). Furthermore, DFE treatment significantly alleviated the damage to ARPE-19 cell viability caused by OXA chemotherapy, and the efficacy was increased significantly with the increase in DFE concentration (Figure 6B, OXA, *p* < 0.01–0.05). Our results revealed that DFE treatments can potentially provide therapeutic protection in ARPE-19 cells under OXA chemotherapy damage. 

### 3.5. DFE Treatment Alleviates OXA Chemotherapy Damage in the Retinal Tissue of Mice

**Figure 7:**
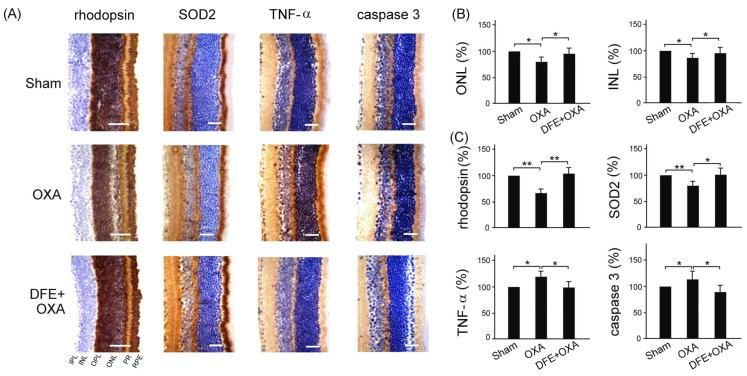
DFE treatment alleviates OXA chemotherapy-induced photopigment deficiency, oxidative stress, inflammation, and apoptosis in the retinal tissue of BALB/c mice. (**A**) IHC staining indicating expressions of rhodopsin, SOD2, TNF-α, and caspase 3 in the retinal tissue of BALB/c mice with treatments of sham, OXA chemotherapy, and DEF + OXA chemotherapy. Scale bar = 150 μm. (**B**) Quantified relative number of cells in outer nuclear layer (ONL) and inner nuclear layer (INL) within the retinal tissue of BALB/c mice with treatments of sham, OXA chemotherapy, and DEF + OXA chemotherapy. (**C**) Quantified relative protein expressions of anti-oxidative stress-related SOD2, inflammation-related TNF-α, and apoptosis-related caspase 3 in the retinal tissue of BALB/c mice with treatments of sham, OXA chemotherapy, and DEF + OXA chemotherapy. Data are shown as mean ± SEM (*n* = 3 for each group, ** *p* < 0.01, * *p* < 0.05, one-way ANOVA followed by Student–Newman–Keuls multiple comparison post tests). IPL: inner plexiform layer; INL: inner nuclear layer; OPL: outer plexiform layer; ONL: outer nuclear layer; PR: photoreceptors; RPE: retinal pigment epithelium.

With the IHC staining assay, we examined the effects of DFE treatment in the retinal tissue of BALB/c mice under sham treatment and OXA chemotherapy damage. Our results showed that DFE treatment can alleviate OXA chemotherapy-induced rhodopsin deficiency, oxidative stress, inflammation, and apoptosis in the retinal tissue of BALB/c mice. (Figure 7A). Rhodopsin is a binding protein composed of retinal and opsin. Also, we observed that the cell thickness of the outer nuclear layer (ONL) and inner nuclear layer (INL) is thinner within the retinal tissue of mice under OXA chemotherapy damage than that of mice under sham and DFE treatments; thus, we quantified the relative number of cells in ONL and INL within the retinal tissue of BALB/c mice with treatments of sham, OXA chemotherapy, and DEF + OXA chemotherapy. Our results showed that mice with treatments of sham and DEF + OXA chemotherapy showed a more relative number of cells in both ONL and INL of the retinal tissue than that of mice with treatments of OXA chemotherapy only (Figure 7B, *p* < 0.05). 

In Figure 7C, we quantified relative protein expressions of anti-oxidative stress-related SOD2, inflammation-related TNF-α, and apoptosis-related caspase 3 in the retinal tissue of BALB/c mice with treatments of sham, OXA chemotherapy, and DEF + OXA chemotherapy. Our results showed that mice with treatments of sham and DEF + OXA chemotherapy showed significantly greater SOD2 expression and significantly lower TNF-α and caspase 3 expressions in the retinal tissue than those of mice with treatments of OXA chemotherapy only (Figure 7C, *p* < 0.01–0.05).

## 4. Discussion

In this study, we used ARPE-19 cells and mouse retinal tissue to study the therapeutic effects of DFE for retinopathy by UV radiation and chemotherapy. The HPLC fingerprint showed that the main active compounds of DFE were moscatilin, gigantol, and chrysotobibenzyl (Figure 2). Our results showed that DFE showed excellent antioxidant capacity (Figure 3). DFE treatment can alleviate a reduction in ARPE-19 cell viability by UV radiation and OXA chemotherapy (Figure 4 and Figure 6); can alleviate damage of the F-actin cytoskeleton structure of ARPE-19 cells by OXA chemotherapy (Figure 5); and can alleviate oxidative stress, inflammation, and apoptosis by OXA chemotherapy (Figure 7). These results demonstrate that DFE can be suitable for the treatment of retinopathy.

The World Health Organization (WHO) defines herbal medicines as labeled finished medicinal products containing active ingredients of plants or combinations of plants [23]. According to the WHO, approximately 80% of the world’s population relies on traditional medicine for their primary health care needs. Even in developed countries, complementary or alternative medicine is growing in popularity [24]. Chinese herbal medicines contain a variety of chemical components that are used for their pharmacological effects on the body. However, the lack of assurance of the safety and efficacy of most herbal products is largely due to insufficient pharmacokinetic, pharmacological, and clinical data [25]. Therefore, we hope that these challenges can be reduced or overcome through our scientific verification.

The most relevant phytochemical elements in dendrobium species include polysaccharides, alkaloids, and polyphenols, and the bioactive components of dendrobium include gigantol, moscatilin, dendrofalconerol A, dendrochrysanene, crispidatin, confusarin, denbinobin, and chrysotobibenzyl [26]. Polysaccharides are among the main active components of dendrobium, exerting antioxidant, anti-inflammatory, and anti-apoptotic effects [27,28,29,30]. In this study, we found that the main active compounds of DFE were moscatilin, gigantol, and chrysotobibenzyl (Figure 2). Moscatilin is a major bibenzyl compound in Dendrobium. Moscatilin from *Dendrobium nobile* Lindley has been considered to protect retinal cells from ischemia/hypoxia by downregulating placental growth factor and upregulating Norrie disease protein [31]. Gigantol is a major polyphenol compound in Dendrobium. Gigantol can protect retinal pigment epithelial cells against high glucose-induced apoptosis, oxidative stress, and inflammation by inhibiting the NF-kB signaling pathway [32]. Chrysotobibenzyl is an aromatic compound. Chrysotobibenzyl extracted from *Dendrobium pulchellum* has been shown to inhibit the growth of lung cancer cells in anchorage-independent conditions [33]. However, the protection effect and underlying mechanisms of chrysotobibenzyl in retinal tissue have not yet been investigated. Whether chrysotobibenzyl can protect or even treat retinopathy will be an interesting topic for us to explore in the future.

Long-term exposure to UV radiation can cause vision deterioration. When the eyes are exposed to UV radiation, reactive oxidative species (ROS) are easily produced, which are associated with retinopathy [34]. Human exposure to UV radiation is associated with many eye diseases, such as cataracts, retinitis pigmentosa, and age-related macular degeneration. UV radiation can initiate free radical formation and ROS production, leading to protein modification, lipid peroxidation, DNA breakdown and mitochondrial dysfunction, and increased apoptotic activity [35]. *Dendrobium nobile* extract has been shown to protect retinal cells from UV-induced oxidative stress damage via the Nrf2/HO-1 and MAPK pathways, which may have a beneficial effect on eye diseases [36]. Our study showed that *Dendrobium fimbriatum* extract has excellent antioxidant capacity and can protect retinal cells from UV damage. We further observed that UV radiation can attenuate F-actin cytoskeleton expressions in ARPE-19 cells, but the attenuation of expressions can be alleviated after treatment with DFE. Our findings thus concluded that DFE can act as a promising therapeutic candidate for UV-induced retinopathy.

Furthermore, our results show that the chemotherapy drug OXA can cause a loss of photoreceptor cells, reducing rhodopsin expression of photoreceptor cells and increasing oxidative stress, inflammation, and apoptosis expressions in mouse retinas. Physicians can pay special attention to this potential retinal toxicity when using chemotherapy drugs, especially OXA. Photoreceptor cells are the first sensory neurons that produce vision. In order to maintain light transmission, photoreceptor cells require a more active metabolism than ordinary neurons to meet high energy demands [37]. Therefore, photoreceptor cells produce a large amount of ROS as an inevitable by-product of metabolism. Once the production of ROS exceeds the intrinsic antioxidant capacity, oxidative stress will occur, resulting in the pathogenesis of visual impairment and retinopathy. Excess ROS are lethal to retinal cells because they can damage cellular proteins, lipids, and DNA and even lead to retinal cell death. Oxidative stress is an important mechanism for OXA-induced retinal toxicity because of the high oxygen consumption in photoreceptors. In addition to reducing antioxidant enzyme activity, OXA also induces oxidative stress by increasing ROS or free radicals and lipid peroxidation [38]. Our study further confirms that DFE can have preventive and alleviating therapeutic effects on retinopathy induced by the cancer chemotherapy drug OXA. The results indeed bring a novel therapeutic application to patients with retinopathy induced by cancer chemotherapy.

In fact, oxidative stress is known to be implicated in the pathology of age-related diseases such as Alzheimer’s, Parkinson’s, Huntington’s, and stroke. Additionally, age-related macular degeneration is a multi-factorial disease, with both environmental and genetic factors playing a role. Environmental causes of age-related macular degeneration remain the subject of discussion. Several risk factors such as cigarette smoking, an excessively high-fat diet, hypertension, and excessive exposure to sunlight have been associated with age-related macular degeneration [39]. As mentioned previously, *Dendrobium nobile* extract can protect ARPE-19 cells from oxidative stress damage by regulating MAPK and Nrf2/HO-1 signaling [36]. Recent evidence also suggests that cGAS-STING signaling plays an important role in mediating inflammation-related diseases such as diabetic retinopathy and age-related macular degeneration [40]. Whether DFE can protect or even treat age-related macular degeneration is a topic of interest to us in the future.

## 5. Conclusions

To sum up our study, we clarified how DFE protects ARPE-19 cells and mouse retinal tissue from damage from UV radiation and chemotherapy. We found that DFE can show excellent antioxidant capacity, alleviate damage of ARPE-19 cells under UV radiation and OXA chemotherapy, and protect mouse retina from damage from the chemotherapy drug OXA by increasing rhodopsin expression and reducing oxidative stress, inflammation, and apoptosis. Thus, we suggest that DFE can be suitable for the treatment of retinopathy.

## Data Availability

Data are available in a publicly accessible repository.

## References

[1] Guymer R.H., Campbell T.G. (2023). Age-related macular degeneration. Lancet.

[2] Maurya M., Bora K., Blomfield A.K., Pavlovich M.C., Huang S., Liu C.H., Chen J. (2023). Oxidative stress in retinal pigment epithelium degeneration: From pathogenesis to therapeutic targets in dry age-related macular degeneration. Neural Regen. Res..

[3] Norval M., Lucas R.M., Cullen A.P., de Gruijl F.R., Longstreth J., Takizawa Y., van der Leun J.C. (2011). The human health effects of ozone depletion and interactions with climate change. Photochem. Photobiol. Sci..

[4] Li Y., Mitchell W., Elze T., Zebardast N. (2021). Association Between Diabetes, Diabetic Retinopathy, and Glaucoma. Curr. Diabetes Rep..

[5] Lechner J., O’Leary O.E., Stitt A.W. (2017). The pathology associated with diabetic retinopathy. Vision Res..

[6] Sung H., Ferlay J., Siegel R.L., Laversanne M., Soerjomataram I., Jemal A., Bray F. (2021). Global Cancer Statistics 2020: GLOBOCAN Estimates of Incidence and Mortality Worldwide for 36 Cancers in 185 Countries. CA Cancer J. Clin..

[7] Schell M.J., McHaney V.A., Green A.A., Kun L.E., Hayes F.A., Horowitz M., Meyer W.H. (1989). Hearing loss in children and young adults receiving cisplatin with or without prior cranial irradiation. J. Clin. Oncol..

[8] Knight K.R., Kraemer D.F., Neuwelt E.A. (2005). Ototoxicity in children receiving platinum chemotherapy: Underestimating a commonly occurring toxicity that may influence academic and social development. J. Clin. Oncol..

[9] Siegel R.L., Miller K.D., Fuchs H.E., Jemal A. (2022). Cancer statistics, 2022. CA Cancer J. Clin..

[10] Chattaraj A., Syed M.P., Low C.A., Owonikoko T.K. (2023). Cisplatin-Induced Ototoxicity: A Concise Review of the Burden, Prevention, and Interception Strategies. JCO Oncol. Pract..

[11] Travis L.B., Fossa S.D., Sesso H.D., Frisina R.D., Herrmann D.N., Beard C.J., Feldman D.R., Pagliaro L.C., Miller R.C., Vaughn D.J. (2014). Chemotherapy-induced peripheral neurotoxicity and ototoxicity: New paradigms for translational genomics. J. Natl. Cancer Inst..

[12] Mesquida M., Sanchez-Dalmau B., Ortiz-Perez S., Pelegrín L., Molina-Fernandez J.J., Figueras-Roca M., Casaroli-Marano R., Adán A. (2010). Oxaliplatin-Related Ocular Toxicity. Case Rep. Oncol..

[13] De Gramont A., Figer A., Seymour M., Homerin M., Hmissi A., Cassidy J., Boni C., Cortes-Funes H., Cervantes A., Freyer G. (2023). Leucovorin and Fluorouracil with or Without Oxaliplatin as First-Line Treatment in Advanced Colorectal Cancer. J. Clin. Oncol..

[14] Simpson D., Dunn C., Curran M., Goa K.L. (2003). Oxaliplatin: A review of its use in combination therapy for advanced metastatic colorectal cancer. Drugs.

[15] Imperia P.S., Lazarus H.M., Lass J.H. (1989). Ocular complications of systemic cancer chemotherapy. Surv. Ophthalmol..

[16] Sun W., Li J., Yan X., Liao L., Li S., Wang X., Xiao C., Shang M., Chao G., Zhou J. (2022). Traditional Chinese Medicine Injections for Diabetic Retinopathy: A Systematic Review and Network Meta-Analysis of Randomized Controlled Trials. J. Integr. Complement. Med..

[17] Ng T.B., Liu J., Wong J.H., Ye X., Wing Sze S.C., Tong Y., Zhang K.Y. (2012). Review of research on Dendrobium, a prized folk medicine. Appl. Microbiol. Biotechnol..

[18] Zhao Y., Son Y.O., Kim S.S., Jang Y.S., Lee J.C. (2007). Antioxidant and anti-hyperglycemic activity of polysaccharide isolated from *Dendrobium chrysotoxum* Lindl. J. Biochem. Mol. Biol..

[19] Yu Z., Gong C., Lu B., Yang L., Sheng Y., Ji L., Wang Z. (2015). Dendrobium chrysotoxum Lindl. alleviates diabetic retinopathy by preventing retinal inflammation and tight junction protein decrease. J. Diabetes Res..

[20] Gong C.Y., Yu Z.Y., Lu B., Yang L., Sheng Y.C., Fan Y.M., Ji L.L., Wang Z.T. (2014). Ethanol extract of *Dendrobium chrysotoxum* Lindl ameliorates diabetic retinopathy and its mechanism. Vascul. Pharmacol..

[21] Zhang Q., Li J., Luo M., Xie G.Y., Zeng W., Wu Y., Zhu Y., Yang X., Guo A.Y. (2020). Systematic Transcriptome and Regulatory Network Analyses Reveal the Hypoglycemic Mechanism of *Dendrobium fimbriatum*. Mol. Ther. Nucleic Acids..

[22] Luo A., Fan Y. (2011). In vitro antioxidant of a water-soluble polysaccharide from Dendrobium fimhriatum Hook.var.oculatum Hook. Int. J. Mol. Sci..

[23] World Health Organization (1993). Research Guidelines for Evaluating the Safety and Efficacy of Herbal Medicines.

[24] World Health Organization (2023). Integrating Traditional Medicine in Health Care.

[25] Parveen A., Parveen B., Parveen R., Ahmad S. (2015). Challenges and guidelines for clinical trial of herbal drugs. J. Pharm. Bioallied Sci..

[26] Oskouei Z., Ghasemzadeh Rahbardar M., Hosseinzadeh H. (2023). The effects of Dendrobium species on the metabolic syndrome: A review. Iran. J. Basic Med. Sci..

[27] Chen H., Shi X., Zhang L., Yao L., Cen L., Li L., Lv Y., Wei C. (2022). Ultrasonic extraction process of polysaccharides from *Dendrobium nobile* Lindl.: Optimization, physicochemical properties and anti-inflammatory activity. Foods.

[28] Luo A., He X., Zhou S., Fan Y., He T., Chun Z. (2009). In vitro antioxidant activities of a water-soluble polysaccharide derived from *Dendrobium nobile* Lindl. extracts. Int. J. Biol. Macromol..

[29] Zhang S., Tu H., Zhu J., Liang A., Huo P., Shan K., He J., Zhao M., Chen X., Lei X. (2020). *Dendrobium nobile* Lindl. polysaccharides improve follicular development in PCOS rats. Int. J. Biol. Macromol..

[30] Lei X., Huo P., Xie Y.J., Wang Y., Liu G., Tu H., Shi Q., Mo Z.C., Zhang S. (2022). *Dendrobium nobile* Lindl polysaccharides improve testicular spermatogenic function in streptozotocin-induced diabetic rats. Mol. Reprod. Dev..

[31] Chao W.H., Lai M.Y., Pan H.T., Shiu H.W., Chen M.M., Chao H.M. (2018). *Dendrobium nobile* Lindley and its bibenzyl component moscatilin are able to protect retinal cells from ischemia/hypoxia by dowregulating placental growth factor and upregulating Norrie disease protein. BMC Complement. Altern. Med..

[32] Chen Y., Zhao T., Han M., Chen Y. (2023). Gigantol protects retinal pigment epithelial cells against high glucose-induced apoptosis, oxidative stress and inflammation by inhibiting MTDH-mediated NF-kB signaling pathway. Immunopharmacol. Immunotoxicol..

[33] Chanvorachote P., Kowitdamrong A., Ruanghirun T., Sritularak B., Mungmee C., Likhitwitayawuid K. (2013). Anti-metastatic activities of bibenzyls from *Dendrobium pulchellum*. Nat. Prod. Commun..

[34] Ivanov I.V., Mappes T., Schaupp P., Lappe C., Wahl S. (2018). Ultraviolet radiation oxidative stress affects eye health. J. Biophotonics.

[35] Glickman R.D. (2011). Ultraviolet phototoxicity to the retina. Eye Contact Lens..

[36] Hsu W.H., Chung C.P., Wang Y.Y., Kuo Y.H., Yeh C.H., Lee I.J., Lin Y.L. (2022). *Dendrobium nobile* protects retinal cells from UV-induced oxidative stress damage via Nrf2/HO-1 and MAPK pathways. J. Ethnopharmacol..

[37] Léveillard T., Sahel J.A. (2017). Metabolic and redox signaling in the retina. Cell Mol. Life Sci..

[38] Liu C.Y., Francis J.H., Brodie S.E., Marr B., Pulido J.S., Marmor M.F., Abramson D.H. (2014). Retinal toxicities of cancer therapy drugs: Biologics, small molecule inhibitors, and chemotherapies. Retina.

[39] Chakravarthy U., Wong T.Y., Fletcher A., Piault E., Evans C., Zlateva G., Buggage R., Pleil A., Mitchell P. (2010). Clinical risk factors for age-related macular degeneration: A systematic review and meta-analysis. BMC Ophthalmol..

[40] Zhou L., Ho B.M., Chan H.Y.E., Tong Y., Du L., He J.N., Ng D.S.C., Tham C.C., Pang C.P., Chu W.K. (2023). Emerging Roles of cGAS-STING Signaling in Mediating Ocular Inflammation. J. Innate Immun..

